# Genome-wide association mapping uncovers sex-associated copy number variation markers and female hemizygous regions on the W chromosome in *Salix viminalis*

**DOI:** 10.1186/s12864-021-08021-2

**Published:** 2021-10-02

**Authors:** Henrik R. Hallingbäck, Pascal Pucholt, Pär K. Ingvarsson, Ann Christin Rönnberg-Wästljung, Sofia Berlin

**Affiliations:** 1 Department of Plant Biology, Uppsala BioCenter, Swedish University of Agricultural Sciences and Linnean Center for Plant Biology, Box 7080, SE-750 07 Uppsala, Sweden; 2 Present Address: Skogforsk (The Forestry Research Institute of Sweden), Uppsala Science Park, SE-751 83 Uppsala, Sweden

**Keywords:** Sex determination, Salix viminalis, Female heterogamety, Association mapping, GWAS, Paralogous loci, Copy number variation, Sex specific markers

## Abstract

**Background:**

Sex chromosomes are in some species largely undifferentiated (homomorphic) with restricted sex determination regions. Homomorphic but different sex chromosomes are found in the closely related genera *Populus* and *Salix* indicating flexible sex determination systems, ideal for studies of processes involved in sex chromosome evolution. We have performed genome-wide association studies of sex and analysed sex chromosomes in a population of 265 wild collected *Salix viminalis* accessions and studied the sex determining locus.

**Results:**

A total of 19,592 markers were used in association analyses using both Fisher’s exact tests and a single-marker mixed linear model, which resulted in 48 and 41 sex-associated (SA) markers respectively. Across all 48 SA markers, females were much more often heterozygous than males, which is expected if females were the heterogametic sex. The majority of the SA markers were, based on positions in the *S. purpurea* genome, located on chromosome 15, previously demonstrated to be the sex chromosome. Interestingly, when mapping the genotyping-by-sequencing sequence tag harbouring the two SA markers with the highest significance to the *S. viminalis* genomic scaffolds, five regions of very high similarity were found: three on a scaffold that represents a part of chromosome 15, one on a scaffold that represents a part of chromosome 9 and one on a scaffold not anchored to the genome. Based on segregation differences of the alleles at the two marker positions and on differences in PCR amplification between females and males we conclude that females had multiple copies of this DNA fragment (chromosome 9 and 15), whereas males only had one (chromosome 9). We therefore postulate that the female specific sequences have been copied from chromosome 9 and inserted on chromosome 15, subsequently developing into a hemizygous W chromosome linked region.

**Conclusions:**

Our results support that sex determination in *S. viminalis* is controlled by one locus on chromosome 15. The segregation patterns observed at the SA markers furthermore confirm that *S. viminalis* females are the heterogametic sex. We also identified a translocation from chromosome 9 to the W chromosome.

**Supplementary Information:**

The online version contains supplementary material available at 10.1186/s12864-021-08021-2.

## Background

Many organisms with genetic sex determination have sex chromosomes that harbour the sex determination (SD) locus. If females are heterogametic and have two different sex chromosomes they are denoted as Z and W (females ZW; males ZZ), whereas if males are heterogametic and have two different sex chromosomes they are denoted as X and Y (males XY; females XX). Sex chromosomes can evolve from a pair of homologous autosomes once one of them acquires a new sex determination factor leading to recombination suppression between these homologous chromosomes. The sex-limited chromosome (W or Y) will thus stop recombining and will consequently evolve independently from its recombining homolog (Z or X). They can also slowly accumulate deleterious mutations, experience translocations, inversions and insertions of repetitive DNA. They can furthermore degenerate over evolutionary time through ectopic recombination, deletions and heterochromatinization [1–3]. In extreme cases, the entire sex-limited chromosome becomes extinct, which for example happened in *Drosophila narragansett*; males X0, females XX [4]. However more often the sex-limited sex chromosome shrink in comparison to its recombining homolog (Z or X). Such heteromorphic sex chromosomes are typically found in many model species such as humans [5], *Silene latifolia* [6] and chicken [7]. But many organisms have homomorphic sex chromosomes that display only low levels of differentiation. These could still be in the initial stages of degeneration as is the situation in wild strawberry [8, 9], garden asparagus [10] and papaya [11, 12]. However, in a recent review, based on empirical data, it was shown that the size of the sex determining region was not correlated with age indicating that the theory predicting homomorphic sex chromosomes to be in an early evolutionary stage does not hold [13]. In some clades, closely related species have different SD loci due to rapid and repeated turnover of sex chromosomes across short evolutionary timescales [14–16]. Sex chromosome turnover can arise when new SD genetic factors evolve, by duplication or translocation of the original genetic factor to new genomic locations, or due to the fusion of autosomes to existing sex chromosomes [17]. These turnover events can initialize the evolution of novel sex chromosomes that provide a unique possibility for the analysis of the initial steps of sex chromosome evolution [18, 19].

Willows (*Salix* spp.) and poplars/aspens (*Populus* spp; henceforth called *poplars* for simplicity) are sister lineages in the Salicaceae plant family. They are (together with several other lineages in this family) dioecious and thus exhibiting male and female flowers on separate individuals [20]. The most parsimonious explanation is therefore that dioecy in this clade is ancient and evolved prior to the divergence of the dioecious lineages. This should then have occurred more than 45 million years ago, which is the approximate divergence time of willows and poplars [21, 22]. In plants, one early theory is that sex chromosomes are expected to evolve when separate sexes evolve from co-sexual ancestors [23]. According to that assumption and since dioecy is ancient in the *Populus/Salix* clade, conserved sex chromosome in willows and poplars would be expected. This is however not the case as the sex chromosomes in both willows and poplars are homomorphic with SD loci on different chromosomes. Emerging knowledge indicate a flexible sex determination system with frequent sex chromosome turnover within the two genera [13, 24]. For the majority of investigated poplar species, chromosome 19 is the sex chromosome [25–35] but in *P. euphratica* chromosome 14 is the sex chromosome [24]. In willows chromosome 15 is the sex chromosome in many of the investigated species [36–43], although chromosome 7 and 14 also have been identified as the sex chromosomes for *S. dunii* and *S. nigra* respectively [44, 45]. Furthermore, most poplars are male heterogametic while the majority of the studied willows are female heterogametic but Z-W heterogamy exist in poplars [24] and X-Y heterogamy have been identified in willows [45]. Taken together, these studies suggest a fast and dynamic sex chromosome evolution in the *Salix* and *Populus* genera. New studies have also suggested a common underlying genetic mechanism for sex determination that includes a cytokinin response regulator [24, 31].

In an earlier study of the sex chromosomes in *Salix viminalis* [41], we used DNA-seq and RNA-seq data from females and males and investigated the level of differentiation in the SD locus compared to the autosomes. We did not find any evidence for genetic decay at the sequence level in the SD locus but we found that the Z and W homologs have started to differentiate as females were observed to have higher single nucleotide polymorphism (SNP) densities than the males in the SD region indirectly suggesting that recombination was recently halted between Z and W. We therefore concluded that the sex chromosomes in *S. viminalis* are among the youngest studied thus far [41]. Furthermore, a major genome assembly effort undertaken for *S. viminalis* allowed additional scrutiny of the SD locus and concluded that its size and divergence was, so far, limited [46]. This would again, imply rapid and recent turnover of sex chromosomes in willows and poplars, making this system ideal for studies of early processes driving sex chromosome evolution.

Previous studies of the sex chromosomes in *S. viminalis*, including mapping of the locus [39, 40] and the previously mentioned DNA- and RNA-seq analyses [41] were done in bi-parental pedigree populations and in a very limited set of unrelated individuals respectively. This limits the interpretation of the existing sex chromosome system as we might have missed some loci that segregate in natural populations with greater genetic diversity. In contrast, in this study we investigate marker-sex associations in a highly diverse genetic material consisting of 265 presumably unrelated accessions, collected from various localities in Europe by conducting genome-wide association mapping (GWAS) [47, 48]. GWAS has also been conducted previously for *S. viminalis* [49] and can be quite efficient in finding causative mutations for interesting traits. This is because in populations of largely unrelated individuals, the correlation between loci (linkage disequilibrium, LD) is expected to not stretch far throughout the physical genome due to extensive number of recombination events that have occurred during the many generations since the latest common ancestor [50, 51]. In *S. viminalis* there are previous indications of an LD extent to about 4000 bp (*r*^2^ ≥ 0.2) [52] but that was for a population that was considerably less diverse genetically than the population of this study. Associated markers are therefore likely to be located very close to the gene that is affecting the trait of interest. With this experimental setup we were able to pinpoint genomic regions underlying the sex trait with great precision and to conduct an analysis of the SD locus to identify markers closely linked to sex in *S. viminalis*.

## Results

### Several markers were significantly associated with sex

**Fig. 1:**
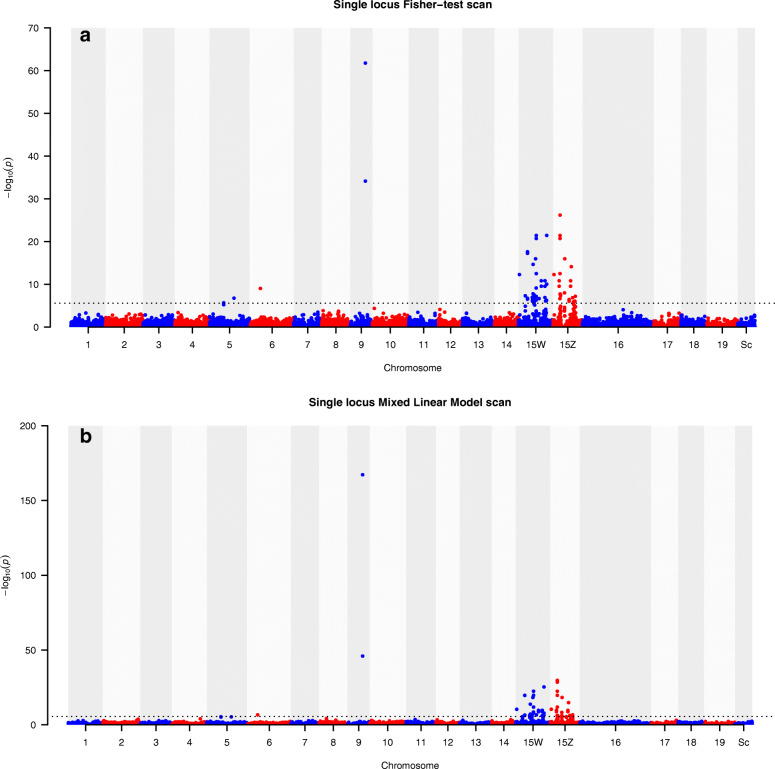
Manhattan plot showing log_10_ (*p*)-values of 19,592 marker-sex associations performed by serial Fisher-tests (**a**) and Mixed linear model (**b**). The 19,592 tested GBS-markers are depicted as mapped to the genome assembly of *S. purpurea v5.1*. For chromosome 15 the markers are plotted against 15W and 15Z separately, Sc: scaffolds. A significance threshold for an experimentwise error rate of 0.05 (Bonferroni correction) is shown as a black line

Sex was morphologically assessed in 265 accessions, and 162 were scored as females (61 %) and 103 as males (39 %). Accession collections from all geographical sampling regions (Eastern Europe, Western Europe, Sweden and Russia) comprised both males and females. We performed a GWAS based on non-parametric Fisher’s exact tests to identify associations between the genotyping by sequencing (GBS) derived markers and the sex phenotype of all scored individuals. However, in order to account for possible genetic structure, we also repeated the GWAS between markers and sex by using mixed linear model (MLM)-analyses including population structure and kinship-based genetic variance components. In total, 48 and 41 significant associations (Bonferroni corrected *p* < 0.05) were identified using Fisher’s exact tests and MLM respectively (Fig. 1) and 39 of the significant associations were consistent across methods. The two methods thus yielded similar results, suggesting that population structure did not significantly impact the associations. Given that the sex trait is not a quantitative character, we proceed with the associations detected by Fisher’s exact tests only.

### Segregation of associated markers fits with ZW sexual system

Across the 48 sex associated (SA) markers, females appeared to be *a*/*b* heterozygous (where *b* is defined as the rare allele) much more frequently than the males, which were mostly *a*/*a* homozygous (see Supplementary file 1). This would be expected if the rare *b*-allele was located in the W chromosome of a ZW sex chromosome system where females are heterogametic (ZW) and males are homogametic (ZZ). Notably, a close to perfect association between genotype and sex was observed for the most significantly associated marker (S1_198703286, *p =* 1.7*10^–62^) as all but three females were scored as *a*/*b*-heterozygous and the *b-*allele was found in two males only (Supplementary file 1). Therefore, the *b*-allele at this locus is likely linked to a female specific W-chromosomal region that shows no, or very little, recombination with its Z-homolog. For most of the other associated markers, the presence of the *b-*allele was entirely (18 of 48 markers), or almost entirely (17 of 48 markers) exclusive to females although the *b-*allele was not necessarily present in all females. One likely explanation for the latter observation is that different W-chromosomes segregate in the population, some of which may share the common *a-*allele with the Z-chromosomes. In contrast, e.g. markers 6 and 29 in Supplementary file 1 for instance showed a very different pattern where homozygosity for allele *b* was almost exclusive to males. This could be explained by two different alleles (*a* and *b*) located on the Z chromosomes while the female limited W homolog only had the common allele (*a*). Females would then have a disproportionately higher frequency of the *a-*allele in comparison to males.

### Majority of sex-associated markers were located on chromosome 15

Based on the genomic positions of the SA markers in the *S. purpurea* genome assembly (v5.1), the majority were located on chromosome 15 (39 of 48) (Fig. 1). Among those, 18 SA markers were found to be located on chromosome 15W, 8 on chromosome 15Z and 13 markers were equally likely to be located on 15W and 15Z. Interestingly, the two SA markers (1 and 2 in Supplementary file 1) with the overall highest significance, were located on the same GBS sequence tag (17 bp apart) which, in turn, was positioned on *S. purpurea* chromosome 9. These SA markers are henceforth referred to as SA marker 1 and SA marker 2. Another three less significant markers were located on *S. purpurea* chromosomes 5 and 6 and additional four markers could not be confidently mapped to any specific location in the *S. purpurea* genome assembly v5.1.

As an alternative to associating markers directly to the *S. purpurea* assembly, we also linked our SA markers to scaffolds in the *S. viminalis* assembly [46] and thereafter those scaffolds were *per se* aligned to the *S. purpurea* genome assembly v5.1. All 48 SA markers mapped to 19 different *S. viminalis* scaffolds and as many as ten SA markers mapped to one single scaffold (Table 1). According to the genetic linkage map markers [40], six of the scaffolds, containing 32 of the 48 SA markers (67 %), were positioned on *S. viminalis* chromosome 15. Moreover, nine of the scaffolds, containing 36 of the 48 SA markers (75 %), were found to be aligned with the female-specific chromosome 15W genome assembly of *S. purpurea* (v5.1) (Table 1). The positions of all chromosome 15 scaffolds containing SA markers were mapped within the genetic position range of 57.2–70.4 cM, encompassing the position where the morphological marker “sex” was mapped (60.7 cM) according to Pucholt et al. [40].

**Table 1:** Characteristics and positions of *Salix viminalis* scaffolds that contain significantly sex-associated markers (Bonferroni-corrected *p* < 0.05, Fisher’s exact test)

*S. viminalis* scaffold^a^	Scaffold length (bp)	Chr based on *S. viminalis* linkage mapping^b^	Center position in *S. viminalis* linkage map, if on Chr15 (cM)^b^	Chr in *S. purpurea v5.1*,^c^	Number of sex-associated markers
0702	768,110	Chr15	69.0	Chr15W	10
0535	622,702	Chr15	57.2	Chr15W	9
1391	539,124	Chr15	70.4	Chr15W	5
1541	2553,872	Chr15	68.0	Chr15W	4
1658	116,437	NA	NA	NA	3
0302	814,410	Chr15	58.0	Chr15W	2
0724	541,616	NA	NA	Chr15W	2
1236	355,459	Chr15	67.6	Chr15W	2
0779	3,309,791	Chr05	NA	Chr15W	1
1114	392,477	NA	NA	Chr15W	1
1491	291,271	NA	NA	Chr16	1
1649	120,404	NA	NA	NA	1
1730	2,005,520	Chr14	NA	Chr14	1
1839	54,690	NA	NA	NA	1
2018	1,314,944	Chr11	NA	Chr11	1
2040	3,822,569	Chr02	NA	Chr02	1
2041	1,625,512	Chr17	NA	Chr17	1
2173	26,877	NA	NA	NA	1
2218	1,126,343	Chr14	NA	Chr14	1
1112^d^	7,166,859	Chr09	NA	Chr09	0

^a^Abbreviated names for scaffolds in the *S. viminalis* genome assembly are used in the table, e.g. ENA|CAADRP010000702|CAADRP010000702.1 will be called 0702 etc

^b^Majority of map markers. Linkage mapping markers described in [40]

^c^Position of that chromosome/scaffold in the *S. purpurea* genome v5.1, majority. For each hit on chromosome 15W, there were always also slightly less significant hits found on chromosome 15Z

^d^Scaffold 1112 is included here since it contains a highly similar secondary blast hit for the tag sequence of the highest ranking SA markers

The tag sequence of the most significant GWAS association, SA marker 1 (S1_198703286), had its best match on the *S. viminalis* genome assembly scaffold 1114 (abbreviation of ENA|CAADRP010001114|CAADRP010001114.1), that did not harbour any other SA markers. However, SA marker 1 also showed three high-scoring secondary matches in the scaffold 0535 (Table 2), which furthermore harbored eight additional SA markers (Table 1). Scaffold 0535 was also the best match for SA marker 2 (S1_198703269) which was the second-most significant association detected in the GWAS. Both scaffolds 1114 and 0535 were associated to chromosome 15W *S. purpurea* genome v5.1 and scaffold 0535 was furthermore aligned with chromosome 15 in *S. viminalis* based on four linkage mapping markers (Table 1). We can thus conclude that scaffold 0535 is most likely located on chromosome 15 in the *S. viminalis* genome and, given its chromosome 15W associations in the *S. purpurea* genome v5.1, it is conceivable that this is true also for scaffold 1114. Taken together, this implies that both SA markers 1 and 2 are in fact located in chromosome 15 in *S. viminalis* despite the initial location at chromosome 9 observed from the initial GWAS results. Another five scaffolds with five less significant markers were potentially part of chromosome 2, 11, 14, and 17 according both to *S. viminalis* map markers and based on their *S. purpurea* v5.1 genome associations (Table 1). Note that outright mapping disagreement between *S. viminalis* map markers and *S. purpurea* genome v5.1 only occurred once for scaffold 0779 which was located on chromosome 5 according to the former and on chromosome 15 according to the latter. This implies that eventual assembly or mapping problems are limited thus increasing the confidence in the results. When accounting for both *S. viminalis* map marker mapping and *S. purpurea* genome mapping, there were only four scaffolds whose position could not be determined.

**Table 2:** Overview of *S. viminalis* scaffolds and genomic positions with high similarity to the tag sequence of the most significantly associated SA marker 1 (S1_198703286). All blast hits with an e-value < 0.05 are shown

*S. viminalis* scaffold (Chromosome)	Percent identical	Match length	Number of mismatches	Target start	Target end	e-value	Bitscore
1114 (NA)	100	53	0	185,301	185,249	7.00*10^–20^	96.9
0535 (Chr15)	100	53	0	171,930	171,982	7.00*10^–20^	96.9
0535 (Chr15)	100	52	0	78,761	78,710	2.00*10^–19^	95.1
0535 (Chr15)	98.08	52	0	190,880	190,830	1.00*10^–16^	86.0
1112 (Chr09)	96.23	53	2	3,895,886	3,895,938	3.00*10^–17^	87.8

### Detection of a female specific hemizygous genomic region on the W chromosome

Interestingly, when we mapped the GBS sequence tag harbouring SA markers 1 and 2 to the *S. viminalis* genome assembly scaffolds (from a female reference individual), we obtained five high confidence matches (e-value ≤ 10^–17^), three on the previously mentioned scaffold 0535 (already identified to be part of chromosome 15 in both *S. viminalis* and *S. purpurea*), one on 1114 (also indicated to be localized on chromosome 15, at least in *S. purpurea*), and one on a scaffold that harbours map markers from *S. viminalis* chromosome 9 as well as being aligned with chromosome 9 in *S. purpurea* v5.1 (scaffold 1112, Tables 1 and 2). This indicates that a DNA fragment containing these two markers likely exist in multiple copies on chromosome 15 and in one copy on chromosome 9 in the genome of both *S. viminalis* and *S. purpurea*. This observation may also explain why the DNA-tag of SA markers 1 and 2 previously mapped to chromosome 9 in the initial GWAS based on the *S. purpurea* v5.1 genome assembly (Fig. 1).

When the five different sequence copies were aligned it became apparent that they are variable in the positions of SA markers 1 and 2 (Supplementary file 2). At the position of SA marker 1, all three copies on scaffold 0535 as well as the copy on 1114 had an A allele, while the homologous sequence in scaffold 1112 contained a G allele (Supplementary file 2). As males were almost exclusively scored as homozygous G/G for SA marker 1, while females were heterozygous A/G, it is then conceivable that the actual variation scored with this marker reflects copy number variation between males and females. Males would thus have only one locus of the DNA fragment (similar to the allele on scaffold 1112) thus appearing as homozygous, whereas females have more than one locus of this fragment with sequence variation and would appear heterozygous as a result of merged scoring of paralogous GBS tags. A similar pattern was observed for SA marker 2 where two of the three copies on scaffold 0535 contained a T allele while one copy on scaffold 0535 and the copy on scaffolds 1114 and 1112 exhibited the C allele (Supplementary file 2). Males were scored as homozygous C/C at this locus while females were scored mostly heterozygous C/T but in a number of cases also as homozygous C/C. As both SA markers 1 and 2 are in close physical proximity, recombination between them should be rare and their different behaviour might thus be caused rather by copy number variation among females or by scoring problems in the GBS analysis due to the complex marker type. In conclusion, these observations suggest that both females and males have one copy of this DNA fragment on chromosome 9 (scaffold 1112), whereas females have (multiple) copies on chromosome 15 (scaffold 0535 and potentially 1114). We thus raise the hypothesis that the sequence on chromosome 15 would be linked to a female specific W chromosomal region and could be hemizygous in females.

### Testing for copy number variation between females and males using read coverage

**Fig. 2:**
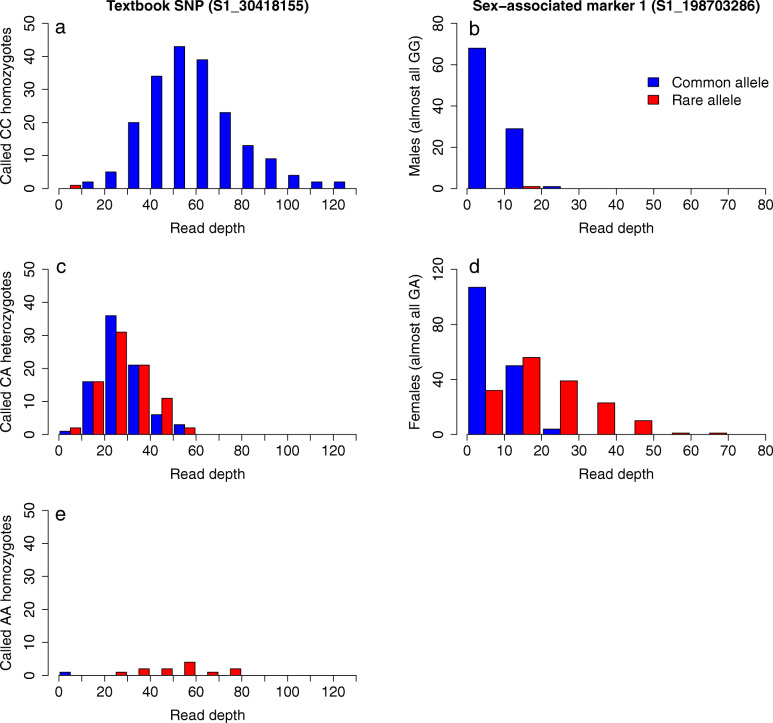
Histogram distribution of a given group of *S. viminalis* accessions by allele-specific read coverage for two selected markers. Each bar signifies a read depth class 10 reads wide. The left column of subplots (**a**, **c**, **e**) shows the distribution of accessions categorized by called genotype across the read depth of a normally behaving marker whereas the right column (**b**, **d**) shows the distribution of sex-specific accessions across the read depth of sex associated marker 1)

In order to test this hypothesis, we compared the read coverage of the two alleles at SA markers 1 and 2 for all accessions (irrespective if they obtained genotypes calls or not) and also compared these with three non-associated high quality GBS markers that adhered to “textbook marker” segregation patterns (100 % genotype completeness, minor allele frequency (MAF) > 5 %, segregation pattern adhering to Hardy-Weinberg equilibrium, c^2^-test, *p* > 0.05). We confidently assume that GBS-derived markers with such a segregation pattern would present accurately called genotypic data reflecting the variation of one single locus only (i.e. a true SNP). For example, the textbook marker SNP S1_30418155 exhibited a high average individual read depth of 60.9 (s.d. 19.4) and the read depth differed very little between females (60.5) and males (61.3) or among differently called genotypes for the marker (61.9, 59.5 and 54.0 for CC, CA and AA respectively). In contrast, the average read depths for the SA markers 1 and 2 were more than three times higher for female accessions (31.9 s.d. 14.1) than for male accessions (9.0 s.d. 5.3) suggesting that the amount of genome sequence being read was systematically higher for females and thus supporting the notion that females exhibit additional sequence copies of the sequence tag comprising SA markers 1 and 2. Moreover, for textbook SNPs, allele-specific read depths in heterozygotes were about halved when compared to the corresponding read depths for homozygote accessions (Fig. 2) illustrating the intuitive principle that the specific read depth for the two alleles in a regular SNP should behave in a dose-dependent and compensatory manner. Again, SA marker 1 did not at all conform to this expectation as the specific read depth of the common G-allele was similar in both sexes (8.8 and 9.6 in males and females respectively) despite most males were homozygous for G while females were GA-heterozygous (Fig. 2). Moreover, in female accessions the A-allele showed an average read depth (22.3 s.d. 11.9) more than twice as high as that of the G-allele in either sex suggesting that the A- and G-allele reads originate, wholly or partially, from different loci. In addition, for SA marker 2, female accessions, whether CC-homozygous or CT-heterozygous, showed a greater average read depth for the common C-allele than did the consistently CC-homozygous males (Supplementary file 3) thus deviating substantially from the dose-dependent and compensatory read depth pattern expected from textbook SNPs. The allele-specific read depth data from SA marker 2 rather indicate that the C-allele reads from females at least partially originate from a different locus than those of the males. Taken together, these observations suggest that the genotype data of SA markers 1 and 2 reflect superimposed observations of at least two paralogous loci of which the first may be largely monomorphic for the common allele in both males and females. The manner in which SA marker 1 A-allele reads were added on top of G-allele reads in females also indicate the A-allele to be strongly linked to one or several elements not present in males or to elements causing null-allele genotypes in males. Again, this points at a female specific hemizygous region at chromosome 15.

### Location of ARR17 homologs in *S. viminalis*

Based on BLASTn searches of ARR17 sequences derived from a number of *Salix* and *Populus* species [31], we identified two homologs in *S. viminalis*. One copy was located on scaffold 0302 which maps close to the sex determining regions on chromosome 15 and which also contained two SA marker according to the GWAS (Fig. 1; Supplementary file 1). The second ARR17 copy was located on scaffold 0502 which maps to chromosome 19.

### Molecular analyses and phylogenetic reconstruction

To further test our hypothesis that the multiple sequence copies on chromosome 15 are linked to a female specific, possibly hemizygous region and that both females and males possess another sequence copy on chromosome 9, we designed a primer pair in conserved regions of the target DNA fragments (Supplementary file 2) and compared the amplification success on an agarose gel (Supplementary file 4) and sequenced the amplification product from two male individuals. As is shown in the multiple alignment of the DNA fragment in Supplementary file 2, the copy in the assembly identified to be part of chromosome 9 contains a 37 bp deletion compared with the copies on chromosome 15 indicating that the sequence can contain length polymorphisms in this locus. Indeed, our hypothesis was supported as multiple bands of different lengths were found in the females (presumably including multiple copies from chromosome 15 and one copy on chromosome 9), whereas the males only exhibited one band (presumably the copy on chromosome 9) (Fig. 1 in Supplementary file 4). A BLAST search revealed two homologs to the amplified male PCR product in the *S. purpurea* genome assembly v.5.1 [43], two homologs in the *S. suchowensis* draft genome assembly but only a homolog on chromosome 9 in the *P. trichocarpa* genome. The phylogenetic reconstruction showed that the *S. purpurea* homolog from chromosome 9, the *S. viminalis* male homologs, the *S. viminalis* sequence from scaffold 1112 and one of the *S. suchowensis* homologs formed a well-supported clade, thus most likely representing sequence copies on chromosome 9 (Supplementary file 4). The *S. viminalis* homologs on chromosome 15 (scaffold 0535) the *S. viminalis* homolog of unknown genomic location (1114) and one of the *S. suchowensis* homologs as well as the *S. purpurea* chromosome 15 W homolog formed the other well-supported clade, thus likely representing the sequence copies on the W-chromosome.

## Discussion

Given all the re-checking and corroborating from various angles undertaken in this study, the results unambiguously support the notion that sex determination in *S. viminalis* is controlled by one locus on chromosome 15. This is a confirmation of our previous findings of single locus sex determination in this willow species. A few weakly associated SA markers were also detected on other chromosomes. Such associations could have been caused by imperfect calling of SNPs based on several paralogous loci which indeed happened despite filtering efforts and which ironically enabled us to identify and further explore sequences in the immediate proximity of the SD locus. It is also possible that current flaws of the *S. viminalis* assembly, as well as possible remaining imperfections in the new *S. purpurea* genome assembly could have caused additional weak sex-marker associations appearing to be located elsewhere in the genome.

The segregation patterns observed at the SA markers furthermore confirmed our previous results that *S. viminalis* females are the heterogametic sex, thus having one Z- and one W-allele at the SD locus, whereas males have two Z-alleles. It should be emphasized that the support for this is not conditioned on whether the apparent greater frequency of called heterozygote females for SA markers is a result of true ZW heterozygosity observed at one single locus or being the result of paralogous loci paired with female hemizygosity (as was observed in some notable cases). Female-specific hemizygosity would *per se* constitute support for females being the heterogametic sex. For many markers the rare allele was confined to females only and was thus likely located on the W chromosome. However, these rare alleles were not always shared by all females indicating that there are likely several alternative alleles within the W chromosome segregating in the population that are tightly linked, but not identical to each other. This observation could reflect the ongoing accumulation of new mutations in the non-recombining region that have not yet been lost or fixed in the population. For some significantly associated markers, the rare and putatively W-linked allele was found also in males, which is a pattern that does not conform to strict non-recombining Z and W sex chromosomes. One possible explanation to this pattern is that very rarely, recombination between the Z and W homologs do happen. This is particularly likely if the sex chromosomes are similar, which is the case if they are very young and in the early phases of differentiation. As we have previously established that the sex chromosomes in this species are among the youngest studied thus far and that the SD locus is narrow, meaning that the sex chromosomes are similar over most of their lengths [41], this explanation cannot be ruled out. In a few cases the sampled marker was highly variable in both males and females thus also making variation in the Z chromosome conceivable (e.g. SA marker 6 and 29 in Supplementary file 1). Finally, some markers displayed segregation patterns which could be an indication of allelic incompatibility [40] or other forms of selection or they might be similar to the case demonstrated for SA markers 1 and 2 (Supplementary file 1) representing compounded reads from two or more paralogous regions.

Despite the recent cessation of recombination between the Z and the W homologous regions close to the SD locus in chromosome 15 in *S. viminalis*, we discovered female specific, and thus W-linked DNA fragments in, or very close to, the SD locus, suggesting that the Z and W have started to become structurally differentiated. Another interesting finding was that this particular DNA fragment was present in multiple copies on the W chromosome with no homologs on the Z chromosome, suggesting that we are dealing with a hemizygous genomic region with repetitive DNA. Therefore, any genetic markers in this region would be present in females only. Indeed, previous studies in *S. viminalis* identified female specific markers [53, 54], of which ‘SCAR354’ was later positioned on chromosome 15 in the taxonomically related *P.trichocarpa* genome [55]. Possibly these markers are located in a hemizygous region in the SD locus on chromosome 15 in willows.

An intriguing observation was that one copy of the DNA fragment was present on chromosome 9 in both females and males in addition to the female specific copies on chromosome 15. Since a homolog of the DNA fragment was also found on chromosome 9 in *P. trichocarpa*, this indicates that the ancestral copy is located on chromosome 9. Our results therefore suggest that the W-linked copies were at some point translocated from chromosome 9 in willows. As both a chromosome 9 and chromosome 15 copies also is present in the female *S. suchowensis* draft genome assembly and the female *S. purpurea* v5.1 assembly, this indicates that the translocation happened prior to the divergence of *S. viminalis, S. purpurea* and *S. suchowensis*. Furthermore, previous studies have shown that these closely related species have their SD locus in approximately the same location on chromosome 15 [37, 39] suggesting that these species share the same ZW sex chromosome system. This sex chromosome system likely evolved when a dominant feminizing SD gene invaded a population [18] of a common willow ancestor. The SD gene then spread across the entire ancestral species replacing the old SD system. This SD gene would then be located in the SD locus on chromosome 15 consequently making it equivalent to a W chromosome.

Although we may not have identified the SD gene itself, the aligned scaffold 0535 (chromosome 15) is physically and strongly linked to the SD gene. With the resources at hand, we were unable to determine the point in time when the DNA fragment was translocated from chromosome 9 to 15 and whether the duplications of the fragment leading to the multiple copies happened at the time of translocation or more recently. Furthermore, we cannot know if our DNA fragment has a causative and active function in sex determination. Sex determination in *Salix* and *Populus* has been associated with homologs of the ARR17 gene [31, 43, 56]. We identified two copies of the *ARR17* gene in *S. viminalis*, one located in the vicinity of the sex determining locus on chromosome 15 (see also [43]) whereas the other was located on chromosome 19 which is homologous to the chromosome harboring the sex determining region in *Populus* [31]. When further improvements to the *S. viminalis* assembly are available, it would increase the chances of identifying the role of the hemizygous region reported here and the functional SD gene itself. This would enable further studies on other willow species aiming at understanding how this fascinating ZW sex chromosome system evolved.

## Conclusions

In similarity to several previous studies on *Salix* spp, the results of this study further support sex determination of *S. viminalis* being controlled by one locus on chromosome 15 and that females are the heterogametic sex. In addition, GBS-based association mapping efforts within this study, identified a DNA-based molecular marker (S1_198703286) that co-segregated with sex to such a degree that linkage was observed to be broken in only 5 out of 265 (< 2 %) individuals from a very diverse and highly structured population sampled across large parts of Europe. Curiously, the polymorphism of the sex-associated marker was found to be caused by a copy number variation in its GBS sequence tag where the copies were extremely similar, although not identical, version of the sequence tag. One copy of this sequence tag was located on scaffold 1112 on chromosome 9 and present in all individuals while at least three other copies were located on one particular scaffold 0535 on chromosome 15 and were found only in females. This pattern, in combination with systematic differences in read depth at the sex-associated marker indicates a W-specific hemizygous region at, or close to, the sex-determining locus at chromosome 15 in *S. viminalis*.

## Methods

### Sampling, sexing and DNA extractions

We used plant material from a *S. viminalis* association mapping population that was planted in a field experiment in Pustnäs, south of Uppsala, Sweden (59.80°N, 17.67°E, 25 m AOD), where each accession was represented by six clonal replicates arranged in a randomized complete block design. The population was a collection of wild accessions that originate from the United Kingdom, Sweden, Belgium, Germany, Poland, Czech Republic and Western Russia [49, 57–59]. Young leaves (approximately 200 mg) were sampled from 291 unique accessions and DNA was extracted following a CTAB-protocol described in Pucholt et al. [40], which is a modified protocol from Brunner et al. [60]. Sex was determined by visual inspection of catkins in all flowering accessions (265 in total) in spring 2011, in two of the blocks. Thus, sexing was for most accessions based on two independent clonal replicates.

### Genotyping-by-sequencing

DNA-extracts were genotyped by GBS at the Genomic Diversity Facility, Cornell University, Ithaca, NY, USA. In a manner much similar to that of Elshire et al. [61], the DNA was digested by the *Ape*KI restriction endonuclease, ligated to sample specific barcode adapter sequences and subsequently sequenced on an Illumina sequencing platform (Illumina Inc., San Diego, CA). Sequence reads and polymorphisms were provided by the Genomic Diversity Facility by running the Tassel GBS analysis pipeline v. 3.0.166 [62] using the available genome sequence of the close relative *Salix purpurea* as a mapping reference (*Salix purpurea* v1.0, DOE-JGI, (https://phytozome-next.jgi.doe.gov/info/Spurpurea_v1_0 ). All sequence sites with putative polymorphisms (1,555,794) for all accessions, were merged by Tassel ver. 4.3.7 and were provided to us as Variant Call Format files (VCF, see [49] for details).

For each putative polymorphic site, diploid genotypes were called by finding the maximum likelihood for the observed distribution of haplotype sequence reads [63] provided that the read depth was at least 5 at any particular site and accession. However, raw read counts from the GBS procedure were also extracted for selected markers deemed as interesting for deeper examination, irrespective if the genotype call was performed or not. The polymorphism data produced by GBS was thereafter merged with genotype data from 1,290 SNPs previously developed for this population [58]. Mapping of the old SNPs to the *S. purpurea* genome was performed by BLAST (e-value < 10^− 9^). The polymorphic sites were then filtered based on data completeness and on minor allele frequency (MAF) depending on intended downstream use (see [49] for details).

A more recent genome assembly for *S. purpurea* recently became available (v5.1, https://phytozome-next.jgi.doe.gov/info/Spurpurea_v5_1) which represents a much-improved assemblies, in particular of the putative sex chromosomes [43]. To be able to visualize the GWAS results against this new genome assembly we performed whole-genome alignments between softmasked versions of the v1.0 and v5.1 assemblies using mummer/nucmer v4.0 [64]. The resulting alignments were stringently filtered to avoid redundant mappings and were then converted to .chain files using scripts from the ‘crossmap-workflow’ package (available at https://github.com/soybase/crossmap-workflow). The .chain files were finally used to lift the GWAS results from v1.0 to v5.1 coordinates using ‘CrossMap’ [65] (available at http://crossmap.sourceforge.net/).

### Association mapping

In brief, the markers used for the association mapping of sex were required to be biallelic, have clear genotypic calls in at least 75 % of the accessions and MAF > 5 %. In all, the fully filtered sex-association mapping dataset comprised 19,592 polymorphic markers. Details are described in Hallingbäck et al. [49] but for the association mapping of sex, no efforts were made to impute missing values in the dataset.

In order to correctly conform to the dioecious and dichotomous behaviour of sex in *S. viminalis*, association mapping was performed by Fisher’s exact tests in R making separate tests for each marker (H_0_: independence of genotype and sex). Marker-sex associations were called significant based on an experiment-wise error rate of 5 % (i.e. Bonferroni-corrected *p-*value < 0.05). However, in order to account for the unlikely possibility that sex could be influenced by population structure, individual relatedness or by polygenic inheritance, we also performed single-marker MLM association analyses where discrete population structure and a random term coupled to a kinship matrix, were included as model components [66]. MLM analyses were performed in a single association scan using the software TASSEL v. 5.2.29 [67] and the program was set to re-estimate the kinship variance component for each single-marker analysis. Population structure components and a kinship matrix necessary for the MLM analyses were inferred [49] using the markers described previously. In most cases, the same marker filtering criteria was used as for the association mapping itself but, for the calculation of structure components and the kinship matrix, markers were required to show 95 % genotyping completeness and to exhibit a MAF at only 1 % or higher. In total 19,243 markers were deemed usable according to these criteria for the kinship calculation.

### Anchoring of *Salix viminalis* genome assembly and sex markers to *S. purpurea* genome

We downloaded the *S. viminalis* genome assembly as published by Almeida et al. [46] from ENA using the accession CAADRP010000000. To obtain an approximate position in the *S. viminalis* genome of the scaffolds harbouring the sex-associated markers (SA markers) we used a set of 1,976 markers whose genomic positions were known on a saturated linkage map previously developed based on a biparental progeny population (so called *map markers*) [40]. The genome scaffolds were assumed to be connected to the genomic location of the map markers that were identified within the scaffold sequence by BLASTn search (e-value < 10^–10^). Similarly, we also aligned the *S. viminalis* scaffolds to the newest version of the *S. purpurea* genome assembly (v5.1, [43]) using mummer/nucmer v4.0 ([64] to identify the location in *S. purpurea* of scaffolds carrying sex-linked markers in *S. viminalis*. Finally, sex determination regions in multiple Salicaceae species have been associated with the presence of homologs of the ARR17 gene. We therefore obtained sequences of ARR17 genes from *Salix purpurea*, *Populus tremula* and *P. trichocarpa* [31] (sequences available at https://static-content.springer.com/esm/art%3A10.1038 %2Fs41477-020-0672-9/MediaObjects/41477_2020_672_MOESM5_ESM.txt) and used BLASTn to identify the location of these genes in *S. viminalis*, using a stringent cutoff e-value < 10^–10^.

### Molecular and phylogenetic analyses

A PCR primer pair: Sf (forward, TATCTGGTCCTGATCGATAC) and Sr (reverse, GCAATCACTAAAGCTTGCTG) was designed in conserved regions of the target DNA fragment (see Supplementary file 2). PCRs were run on Biorad MyCycler (Bio-Rad Laboratories, Inc., Hercules, CA, USA) using an initial denaturation step of 95 °C for 5 min, 30 cycles of extension with 30 s of denaturation at 95 °C, 30 s of annealing with an initial touchdown phase of 1 °C per cycle from 60 to 55 °C for the remaining cycles and 90 s of extension at 72 °C, finished by a final extension phase of 10 min at 72 °C. We used the AmpliTaq Gold polymerase (Thermo Fisher Scientific Inc, Waltham, MA, USA) with standard buffer concentrations and DNA from two female accessions (78,183 and 78,021) and two male accessions (81,084 and T76) from the association mapping population. The PCR amplifications were analysed on a 1.5 % agarose gel and by Sanger sequencing of the PCR products from the two males (Macrogen Inc., Seoul, Korea). Sequences were analysed and edited using the SeqMan Pro module in the Lasergene 12 package (DNASTAR Inc., Madison, WI, USA). Homologous sequences to the male consensus PCR fragments were identified by BLASTn in the *S. viminalis* genome assembly and in the female *S. purpurea* v5.1, the female *S. suchowensis* v2.0 (http://115.29.234.170/willow) draft genome assemblies and the *P. trichocarpa* genome. The homologs were then aligned, and a phylogenetic tree was reconstructed (for details on methodology see Supplementary file 4).

## Supplementary Information

**Additional file 1.**

**Additional file 2.**

**Additional file 3.**

**Additional file 4.**

## Data Availability

In this work we used the *Salix viminails* genome as it is deposited at the European Nucleotide Archive, ENA ( https://www.ebi.ac.uk/ena/browser/view/CAADRP010000000) with the accession CAADRP010000000 within the study PRJEB31619. The raw, unfiltered set of SNPs generated by genotyping-by-sequencing were deposited at the Zenodo open data archive and are available at:10.5281/zenodo.2607520. Phenotypic data is available upon request from corresponding author.

## Abbreviations

GBS: Genotyping by sequencing
GWAS: Genome wide association study
LD: Linkage disequilibrium
MLM: Mixed linear model
MAF: Minor allele frequency
SA: Sex associated
SD: Sex determination
SNP: Single nucleotide polymorphism
