# Supplementary material for: Genome-wide association mapping uncovers sex-associated copy number variation markers and female hemizygous regions on the W chromosome in Salix viminalis
Source: BMC Genomics. 2021 Oct 2;22:710. doi: 10.1186/s12864-021-08021-2 (PMC8487499; doi:10.1186/s12864-021-08021-2)
Supplement: Supplementary file 1 — Additional file 1. [file 12864_2021_8021_MOESM1_ESM.pdf]

Supplementary file 1. Association of genetic markers with phenotypic sex. Chrom: chromosome in the *Salix purpurea* v5.1 genome assembly. Pos: position of the marker in the *S. purpurea* sequence. Sc: scaffold in the *S. viminalis* genome assembly. M[aa], M[ab], M[bb]: number of males of genotype aa, ab, bb. F[aa], F[ab], F[bb]: number of females of genotype aa, ab, bb.

SNP no.	Marker ID	Chrom. on S. purpurea	Pos. (bp), S. purpurea	*p* value	Sc. S. viminalis genome	M[aa]	M[ab]	M[bb]	F[aa]	F[ab]	F[bb]
1	S1_198703286	9	6877165	1,66E-62	1114	79	2	0	0	159	3
2	S1_198703269	9	6877148	7,00E-35	535	81	0	0	39	123	0
3	S1_301105921	15Z	3159693	6,16E-27	702	102	1	0	65	97	0
4	S1_301105798	15Z	3159570	6,16E-27	535	102	1	0	65	96	1
5	S1_417215016	15W	12745730	3,50E-22	702	87	9	3	44	102	12
6	S1_301080503	15W	8017933	3,57E-22	702	10	47	45	60	101	1
6	S1_301080503	15Z	3137695	3,57E-22	702	10	47	45	60	101	1
7	S1_301080348	15W	8017778	1,90E-21	702	100	1	0	77	82	1
7	S1_301080348	15Z	3137540	1,90E-21	702	100	1	0	77	82	1
8	S1_299243316	15W	4049715	2,37E-18	1236	92	10	1	60	99	3
9	S1_299243314	15W	4049713	5,56E-18	1391	91	10	1	60	98	3
10	S1_299575099	15W	7722195	1,04E-16	1391	89	0	0	70	57	0
10	S1_299575099	15Z	5287588	1,04E-16	1391	89	0	0	70	57	0
11	S1_426671503	15W	6578181	2,12E-15	1658	78	0	0	74	61	0
12	S1_458685442	NA	NA	2,57E-15	1658	93	5	0	76	76	0
13	S1_299326588	15Z	8233872	7,34E-15	1391	71	26	4	32	101	22
14	S1_301080349	15W	8017779	3,06E-13	702	100	1	0	102	56	2
14	S1_301080349	15Z	3137541	3,06E-13	702	100	1	0	102	56	2
15	S1_289121195	15W	412302	5,04E-13	535	99	0	0	108	52	0
15	S1_289121195	15Z	412302	5,04E-13	535	99	0	0	108	52	0
16	S1_111879479	15W	11977781	1,45E-11	535	80	0	0	90	44	5
16	S1_111879479	15Z	7933186	1,45E-11	535	80	0	0	90	44	5
17	S1_417215020	15W	12745734	9,27E-11	702	16	75	8	90	59	9
18	S1_111879480	15W	11977782	2,76E-10	535	79	1	0	89	44	6
18	S1_111879480	15Z	2752952	2,76E-10	535	79	1	0	89	44	6
19	S1_301017457	15W	7966167	7,28E-10	535	74	2	0	86	53	0
20	S1_151972333	6	4855562	9,31E-10	2218	97	0	0	117	39	0
21	S1_301246766	15Z	5185757	9,82E-09	1236	99	2	0	117	44	0
22	S1_426671524	15W	6578202	1,61E-08	1658	17	79	0	83	73	0
23	S1_301105872	15Z	3159644	1,79E-08	702	101	2	0	119	43	0
24	S1_291858504	15Z	3327697	2,46E-08	702	100	3	0	116	46	0
25	S1_81210229	15W	7089819	3,06E-08	1649	103	0	0	129	33	0
26	S1_299484971	15W	3051958	4,73E-08	1491	79	6	0	81	52	0
26	S1_299484971	15Z	3399487	4,73E-08	1491	79	6	0	81	52	0
27	S1_301918927	15Z	10075087	6,26E-08	702	103	0	0	130	32	0
28	S1_365440185	15W	7361260	6,26E-08	1541	103	0	0	130	32	0
29	S1_301017495	15W	7966205	6,62E-08	535	12	38	26	45	87	7
30	S1_420059915	15W	6253205	1,09E-07	302	102	0	0	127	30	0
31	S1_111792475	15W	11941269	1,20E-07	779	103	0	0	130	30	0
32	S1_459363879	15W	7168590	1,36E-07	2173	103	0	0	132	30	0
32	S1_459363879	15Z	8815758	1,36E-07	2173	103	0	0	132	30	0

33	S1_121332454	5	11041111	1,69E-07	2040	88	2	0	113	39	4
34	S1_299243551	15W	4049950	1,82E-07	1391	99	1	0	126	35	0
35	S1_298465090	15W	9311326	2,13E-07	302	68	5	0	79	52	0
36	S1_298636583	15Z	3092383	2,19E-07	702	48	27	17	86	4	34
37	S1_375778482	15W	7291536	2,73E-07	724	102	0	0	132	30	0
38	S1_299243470	15W	4049869	2,98E-07	1391	99	1	0	123	34	0
39	S1_302334011	15W	6302333	3,75E-07	1541	103	0	0	132	29	1
39	S1_302334011	15Z	7241906	3,75E-07	1541	103	0	0	132	29	1
40	S1_301017504	15W	7966214	4,68E-07	535	14	39	23	49	84	6
41	S1_315846199	15W	12688516	5,66E-07	1839	94	0	0	128	29	0
42	S1_453754775	NA	NA	7,13E-07	535	44	46	12	29	75	57
43	S1_305855262	15W	12237287	8,25E-07	2041	38	62	0	111	49	0
43	S1_305855262	15Z	9883686	8,25E-07	2041	38	62	0	111	49	0
44	S1_302334017	15W	6302339	8,85E-07	1541	102	1	0	129	32	1
44	S1_302334017	15Z	7241912	8,85E-07	1541	102	1	0	129	32	1
45	S1_295510999	15W	7116225	1,22E-06	1541	98	4	1	116	41	5
45	S1_295510999	15Z	9281757	1,22E-06	1541	98	4	1	116	41	5
46	S1_146539287	NA	NA	1,25E-06	2018	78	0	0	100	24	5
47	S1_375718835	NA	NA	1,70E-06	724	86	0	0	120	28	1
48	S1_116742741	5	6380354	2,11E-06	1730	80	0	0	112	27	0
