# Supplementary material for: Genome-wide association mapping uncovers sex-associated copy number variation markers and female hemizygous regions on the W chromosome in Salix viminalis
Source: BMC Genomics. 2021 Oct 2;22:710. doi: 10.1186/s12864-021-08021-2 (PMC8487499; doi:10.1186/s12864-021-08021-2)
Supplement: Supplementary file 2 — Additional file 2. [file 12864_2021_8021_MOESM2_ESM.pdf]

Supplementary File 2: Multiple sequence alignment of homologous regions. Scaffold CAADRP01000**0535** is linked to the sex determination region of Chr 15 by *S. viminalis* map markers while scaffold CAADRP01000**1112** is linked to Chr 9 and CAADRP01000**1114** could not be assigned to a genomic position. The GBS sequencing tag from which the SA markers 1 and 2 are scored is highlighted and their position is indicated. Sequences labelled "R" are presented as reverse complement of the reference sequence.

Homologous region start

```
1112:3895476-3896017     AGTAGAGAAGATGCGGGAGCTAGAATGAGTGGAAAATATAGTTCTACACAGATGTTAACTTCTGTATGGATCCACTTGTTCTGAA    85
1114:185170-185749-R     AGTAGAGAGGATGGGGAGCTAGAATGAGTGGAAAATATAGTTCTACACAGATGTTAACTTCTGTATGGATCCACTTGTTCTGAA    85
0535:78631-79208-R       TCGGACTCTTACTGTACACTCTGGACATTTGCAATCAATTGTTCTACACAGATGTTAACTTCTGTATGGATCCACTTGTTCTGAA    85
0535:171484-172058       AGTAGAGAGGATGGGGAGCTAGAATGAGTGGAAAATATAGTTCTACACAGATGTTAATTTCTGTATGGATCCACTTGTTCTGAA    85
0535:190750-191328-R     TCGGACTCTTACTGTACACTCTGGACATTTGCAATCAATTGTTCTACACAGATGTTAACTTCTGTATGGATCCACTTGTTCTGAA    85
                                  10        20        30        40        50        60        70        80
```

Primer Svim-SexMarker-Sr

```
1112:3895476-3896017     TCATTTGCAATCACTAAAGCTTG--CTGGGGTAAAAGACCATGTATCTGATATGTCAGATAGTATCCTGCCTGCATAGCGATATT    168
1114:185170-185749-R     TCAATTGCAATCACTAAAGCTTGCTCTGGGGTAAAAGAGCATGTAGCTGATATGTCAGATATTATCCTGCCTGCATAGCGATATT    170
0535:78631-79208-R       TCAATTGCAATCACTAAAGCTTG--CTGGGGTAAAAGAGCATGTAGCTGATATGTCAGATATTATCCTGCCTCTATAACGATATT    168
0535:171484-172058       TCAATTGCAATCACTAAAGCTTG--CTGGGGTAAAAGAGCATGTAGCTGATATGTCAGATATTATCCTGCCTGCATAGCGATATT    168
0535:190750-191328-R     TCAATTGCAATCACTAAAGCTTG--CTGGGGTAAAAGAGCATGTAGCTGATATGTCAGATATTATCCTGCCTCTATAACGATATT    168
                             90        100       110       120       130       140       150       160       170
```

In/Del

```
1112:3895476-3896017     TGATATTGCT-------------------------------------AAAAATTAACCTCATATTACCCTTTCAATGCAGGACGT    216
1114:185170-185749-R     TGATATTGCTAAAAAGTTTAGACTTTAAATTC-TTTTCTGTGACAAAAAAAATTAACCTCATATTACCCTTTCAATGCAGGACGT    254
0535:78631-79208-R       TGATATTGCTAAAAAGTTTAGACTTTAAATTC-TTTTCTGTGACAAAAAAAATTAACCTCATATTACCCTTTCAATGCAGGACGT    252
0535:171484-172058       TGATATTGCTAAAAAGTTTAGACTTTAAATTC-TTTTCTGTGACAAAAAAAATAACCTCATATTACCCTTTCAATGCAGGACGT    252
0535:190750-191328-R     TGATATTGCTAAAAAGTTTAGACTTTAAATTCTTTTTCTGTGACAAAAAAATTAACCCTCATATTACCCTTTCAATGCAGGACGT    253
                                  180       190       200       210       220       230       240       250
```

```
1112:3895476-3896017     CAAGCTGAAGTGCTGGGGATAGAAAAGTGTACGGACCGCTTCCTATTTTCATGTCATTTTGTATCGATCAGGACCAGATAATCTG    301
1114:185170-185749-R     CAAGCTGAAGTGCGCGGGATAGAAAAGTGTACTGGCTGCTTCCTATTTTCATGTCATTTTGTATCGATCAGGACCAGATAATCTG    339
0535:78631-79208-R       CAAGCTGAAGTGCGCGGGATAGAAAAGTGTACTGGCTGCTTCCTATTTTCATGTCATTTTGTATCGATCAGGACCAGATAATCTG    337
0535:171484-172058       CAAGCTGAAGTGCGCGGGATAGAAAAGTGTACTGGCCGCTTCCTATTTTCATGTCATTTTGTATCGATCAGGACCAGATAATCTG    337
0535:190750-191328-R     CAATCTGAAGTGCGCGGGATAGAAAAGTGTACTGGCTGCTTCCTA-TTTCATGTCATTTTGTATCGATCAGGACCAGATAATCTG    337
                             260       270       280       290       300       310       320       330       340
```

Primer Svim-SexMarker-Sf

```
1112:3895476-3896017     CCGCTTCATCCATGAAAACCAGTGGAGAAACCATGCAGCAGTGTATTGTTTCCGGGTCAGGATCCAGGAAGTTATAGTGAGCCCG    386
1114:185170-185749-R     CCGCTTCATCCATGAAAACCAGTGGAGAAACCATGCAGCAGTGTATTGTTTCCCGGTCAGGATCCAGGAAGCTATAGTGAGCCCG    424
0535:78631-79208-R       CCGCTTCATCCATGAAAACCAGTGGAGAAACCATGCAGCAGTGTATTGTTTCCCGGTCAGGATCCAGGAAGCTATAGTGAGCCCG    422
0535:171484-172058       CCGCTTCATCCATGAAAACCAGTGGAGAAACCATGCAGCAGTGTATTGTTTCCCGGTCAGGATCCAGGAAGCTATAGTGAGCCCG    422
0535:190750-191328-R     CCGCTTCATCCATGAAAACCAGTGGAGAAACCATGCAGCAGTGTATTGTTTCCCGGTCAGGATCCAGGAAGCTATAGTGAGCCCG    422
                                  350       360       370       380       390       400       410       420
```

GBS Tag

SA marker 2 *SA marker 1*

```
1112:3895476-3896017     TGCTTCTTCTTCCTTGGA-TATCCACAGCAGGTCGAGTCGTCGTATGCTGGTTGCATGCACCCAGTGGATAATGGCAGCCTGAGT    470
1114:185170-185749-R     TGCTTCTTCTTCCTTGGA-TATCCACAGCAGGTCGAGTCGTCATATGCTGGTTACATGCACCCAGTGGATAATGGCAGCCTGACT    508
0535:78631-79208-R       TGCTTCTTCTTCCTTGGA-TATCCATAGCAGGTCGAGTCGTCATATGCTGGTTACATGCACCCAGTGGATAATGGCAGCCTGACT    506
0535:171484-172058       TGCTTCTTCTTCCTTGGA-TATCCACAGCAGGTCGAGTCGTCATATGCTGGTTACATGCACCCAGTGGATAATGGCAGCCTGACT    506
0535:190750-191328-R     TGCTTCTTCTTCCTTGGATTATCCATAGCAGGTCGAGTCGTCATATGCTGGTTACATGCA-CCAGTGGATAATGGCAGCCTGACT    506
                             430       440       450       460       470       480       490       500       510
```

Homologous region end

```
1112:3895476-3896017     TGAGTGGATCAG-ACA----GATGCCGTTTT------TGCATGATTTTTATACATTTGGATGGAATATAGTAAGTGGTAGGTC    542
1114:185170-185749-R     TGAGTGGATCAG-ATA----GATGCCATTTT------TGCATGATTTTTATACATTTGGATGGAATATAGTAAGTGGTAGGTC    580
0535:78631-79208-R       TGAGTGGATCAG-ATA----GATGCCATTTT------TGCATGATTTTTATACATTTGGATGATTTTTATACAGAGATGTTCC    578
0535:171484-172058       TGAGTGGATAAA-TTAACGGGGATTATTTCTTCAAGTGCGCGGTTTCCTTTCTGTT-------------TCAGTGTCATGTC    575
0535:190750-191328-R     TGAGTGGATCAGAATA----GATGCCATTTT------TGCATGATTTTAATATACATTATGATTTTTTATACAGAGATGTTCC    579
                                  520       530       540       550       560       570       580       590
```
