# Supplementary material for: Genome-wide association mapping uncovers sex-associated copy number variation markers and female hemizygous regions on the W chromosome in Salix viminalis
Source: BMC Genomics. 2021 Oct 2;22:710. doi: 10.1186/s12864-021-08021-2 (PMC8487499; doi:10.1186/s12864-021-08021-2)
Supplement: Supplementary file 3 — Additional file 3. [file 12864_2021_8021_MOESM3_ESM.docx]

**Supplementary file 3**


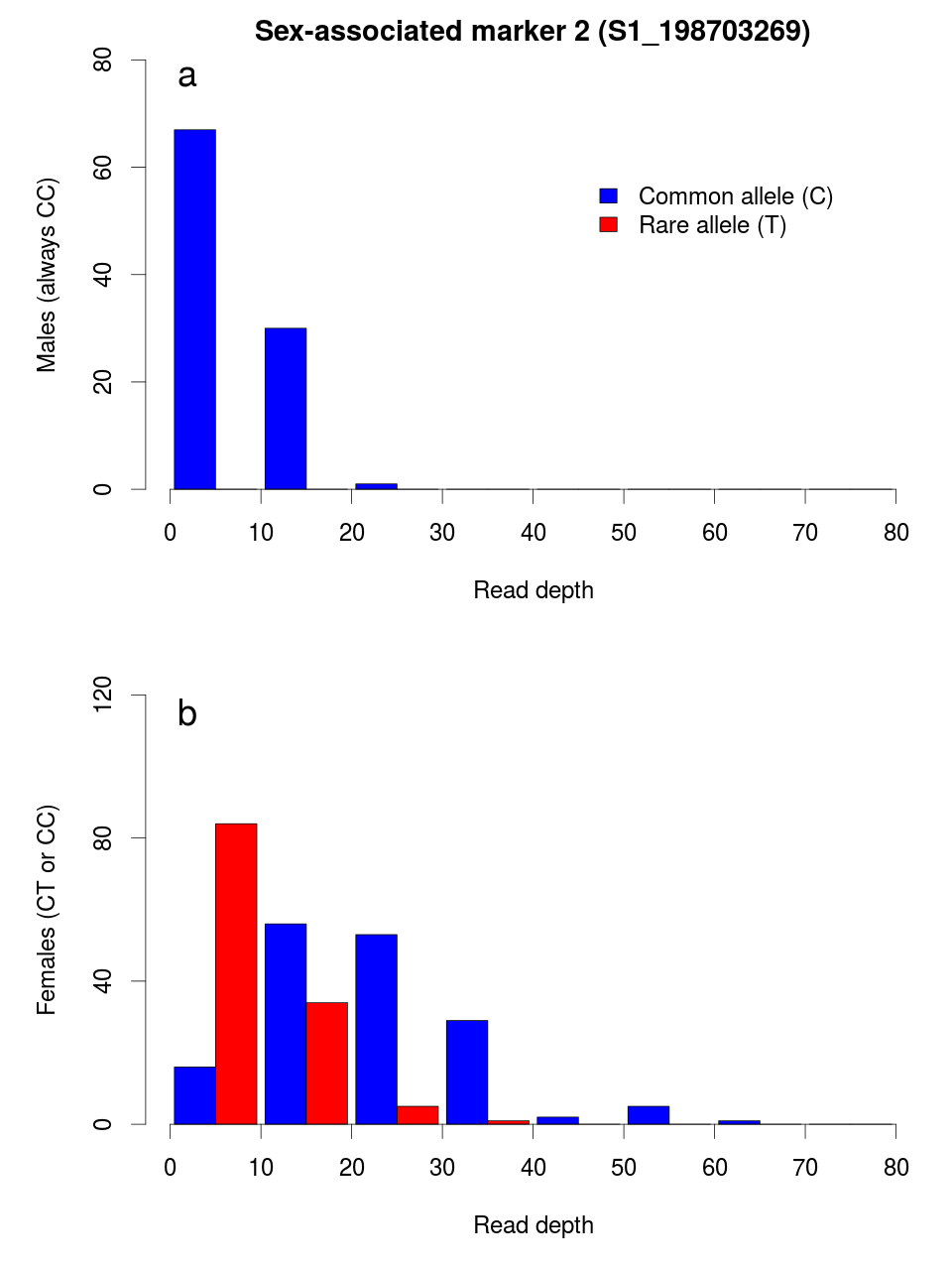


Histogram distribution of a given group of *S. viminalis* accessions by allele-specific read coverage for sex-associated marker 2. Each bar signify a read depth class 10 reads wide. Subplot a shows the histogram for male accessions while subplot b shows the corresponding histogram for females.
