# Supplementary material for: Genome-wide association mapping uncovers sex-associated copy number variation markers and female hemizygous regions on the W chromosome in Salix viminalis
Source: BMC Genomics. 2021 Oct 2;22:710. doi: 10.1186/s12864-021-08021-2 (PMC8487499; doi:10.1186/s12864-021-08021-2)
Supplement: Supplementary file 4 — Additional file 4. [file 12864_2021_8021_MOESM4_ESM.pdf]

Supplementary file 4

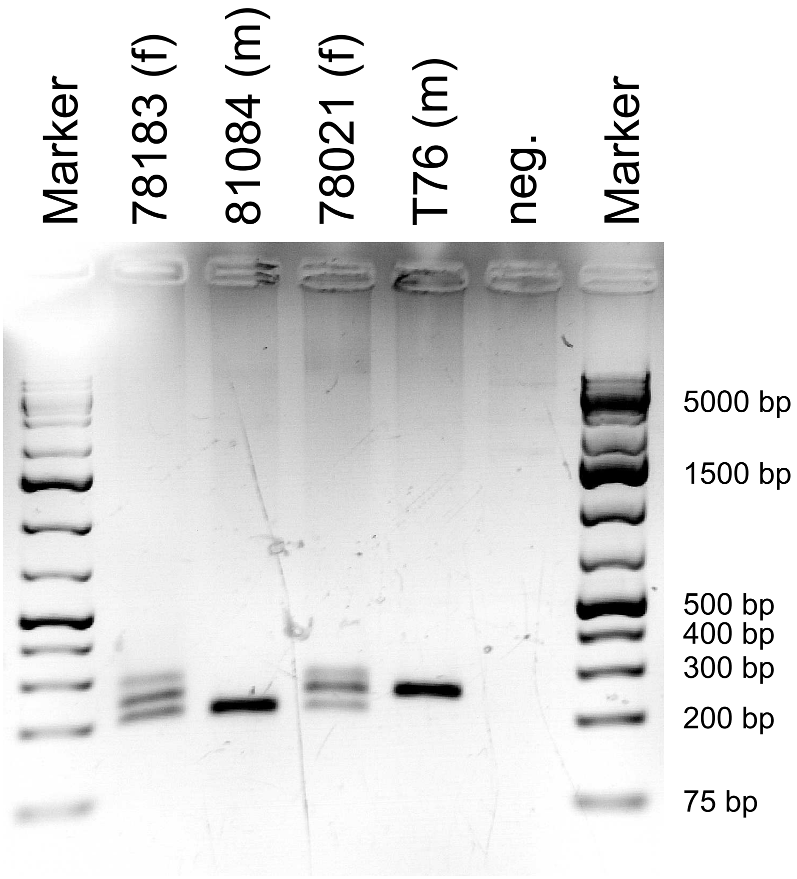

Figure 1: Gel picture of PCR products generated with the primer pair Svim_SexMarker_Sr and Svim_SexMarker_Sf in four individuals. 78183 and 78021 are females, 81084 and T76 are males. From females at least three different PCR products were obtained while male DNA resulted in a single PCR product. Marker: GeneRuler 1kb Plus DNA Ladder (Thermo Fisher Scientific Inc.). neg.: negative control without template DNA.

```
                                                                                    selected
S. viminalis PCR 81084          TTGCT--GGGGTAAAAGACCATGTATCTGATATGTCAGATAGTATCCTGCCTGCA   67
S. viminalis PCR T76            TTGCT--GGGGTAAAAGACCATGTATCTGATATGTCAGATAGTATCCTGCCTGCA   55
S. viminalis 1112               TTGCT--GGGGTAAAAGACCATGTATCTGATATGTCAGATAGTATCCTGCCTGCA  158
S. viminalis 1114               TTGCTCTGGGGTAAAAGAGCATGTAGCTGATATGTCAGATATTATCCTGCCTGCA  160
S. viminalis 0535 79 kbp        TTGCT--GGGGTAAAAGAGCATGTAGCTGATATGTCAGATATTATCCTGCCTCTA  158
S. viminalis 0535 172 kbp       TTGCT--GGGGTAAAAGAGCATGTAGCTGATATGTCAGATATTATCCTGCCTGCA  158
S. viminalis 0535 191 kbp       TTGCT--GGGGTAAAAGAGCATGTAGCTGATATGTCAGATATTATCCTGCCTCTA  158
S. purpurea Chr09               TTGCT--GGGGTAAAAGAGCATGTAGCTGATATGTCAGATAGAATCCTGCCTGCA   79
S. purpurea Chr15W              TTGCT--GGGGTCAAAGAGCATGTAGCTGATATGTCAGATATTATCCTGCCTGCA   79
S. suchowensis scaffold13213    TTGCT--GGGGTAAAAGAGCATGTAGCTGATATGTCAGATATTATCCTGCCTGCA   67
S. suchowensis scaffold04567    TTGCT--GGGGTAAAAGAGCATGTAGCTGATATGGCAGATAGTATCCTGCCTGCA   67
P. trichocarpa Chr09            ----------------------------------TCTAATGGTATCCTGC----A   17
                                 120       130       140       150       160       170

                                      selected
S. viminalis PCR 81084          TAGCGATATTTGATATTGCTAA--------------------AA-A---------   92
S. viminalis PCR T76            TAGCGATATTTGATATTGCTAA--------------------AA-AGTTTAGAGT   89
S. viminalis 1112               TAGCGATATTTGATATTGCTAA--------------------AA-A---------  183
S. viminalis 1114               TAGCGATATTTGATATTGCTAA--------------------AA-AGTTTAGACT  194
S. viminalis 0535 79 kbp        TAACGATATTTGATATTGCTAA--------------------AA-AGTTTAGACT  192
S. viminalis 0535 172 kbp       TAGCGATATTTGATATTGCTAA--------------------AA-AGTTTAGACT  192
S. viminalis 0535 191 kbp       TAACGATATTTGATATTGCTAA--------------------AA-AGTTTAGACT  192
S. purpurea Chr09               CAGCGATATTTGATATTGCTAA--------------------AA--GTTTAGACT  112
S. purpurea Chr15W              TAGCGATATTTGATATTGCTAA--------------------AA-AGTTTAGACT  113
S. suchowensis scaffold13213    TAGCGATATTTGATATTGCTAA--------------------AA-AGTTTAGACT  101
S. suchowensis scaffold04567    CAGCGATATTTGATATTGCTAA--------------------AA--GTTTAGACT  100
P. trichocarpa Chr09            TAGCGATACTTGATATTGCTAACAATATACTGCTTCTTTTAAAAGAGTTTAGGGT   72
                                       180       190       200       210       220

                                                                         selected
S. viminalis PCR 81084          ----------------------------TTAA-CCTCATATTACCCTTTCAATGC  118
S. viminalis PCR T76            TCAAATTCTTT-TCTGTGACAGAAAAAATTAA-CCTCATGTTACCCTTTCAATGC  142
S. viminalis 1112               ----------------------------TTAA-CCTCATATTACCCTTTCAATGC  209
S. viminalis 1114               TTAAATTCTTT-TCTGTGACAAAAAAAATTAA-CCTCATATTACCCTTTCAATGC  247
S. viminalis 0535 79 kbp        TTAAATTCTTT-TCTGTGACAAAAAAAATTAA-CCTCATATTACCCTTTCAATGC  245
S. viminalis 0535 172 kbp       TTAAATTCTTT-TCTGTGACAAAAAAAAATAA-CCTCATATTACCCTTTCAATGC  245
S. viminalis 0535 191 kbp       TTAAATTCTTTTTCTGTGACAAAAAA-ATTAACCCTCATATTACCCTTTCAATGC  246
S. purpurea Chr09               TTAAATTCTTT-TCCGTGACAGAAAAAATCAA-CCTCATATTACCCTTTCAATGC  165
S. purpurea Chr15W              TTAAATTCTTT-TCTGTGACAAAAAAAAAAAA-CCTCATATTACCCTTTCAATGC  166
S. suchowensis scaffold13213    TTAAATTCTTT-TCTGTGACAAAAAAAATTAA-CCTCATATTACCCTTTCAATGC  154
S. suchowensis scaffold04567    TTAAATTCTTT-TCTGTGACAGAAAAAATCAA-CCTCATATTACCCTTTCAATGC  153
P. trichocarpa Chr09            TTAAATTCTTT-TTGGTTGCAGAAAAAATTAA-CTTCACATTACCCTTTCAATGC  125
                                 230       240       250       260       270       280

                                            selected
S. viminalis PCR 81084          ----AGGACGTCAAGCTGAAGT-CTG----GGGATAG-----------AAAAGT-  152
S. viminalis PCR T76            ----AGGACGTCAGGCTGAAGTGCTG----GGGATAG-----------AAAAGT-  177
S. viminalis 1112               ----AGGACGTCAAGCTGAAGTGCTG----GGGATAG-----------AAAAGT-  244
S. viminalis 1114               ----AGGACGTCAAGCTGAAGTGCGC----GGGATAG-----------AAAAGT-  282
S. viminalis 0535 79 kbp        ----AGGACGTCAAGCTGAAGTGCGC----GGGATAG-----------AAAAGT-  280
S. viminalis 0535 172 kbp       ----AGGACGTCAAGCTGAAGTGCGC----GGGATAG-----------AAAAGT-  280
S. viminalis 0535 191 kbp       ----AGGACGTCAATCTGAAGTGCGC----GGGATAG-----------AAAAGT-  281
S. purpurea Chr09               GCGCAGGACGTCAAGCTGAACTGCGCGCGCGGGATAG-----------AAAAGT-  208
S. purpurea Chr15W              ----AGGACGTCAAGCTGAAGTGCGC----GGGATAG-----------AAAAGT-  201
S. suchowensis scaffold13213    ----AGGACGTCAAGCTGAAGTGCGC----GGGATAG-----------AAAAGT-  189
S. suchowensis scaffold04567    GCGCAGGACGTCAAGCTGAACTGCGCGCGCGGGATAG-----------AAAAGT-  196
P. trichocarpa Chr09            ----AGG---TCAAGAGGAAGGACGT----AGGATAGAACTTCAGTAGAAAAGTC  169
                                       290       300       310       320       330

                                            selected                      selected
S. viminalis PCR 81084          ---GTACGG-CCGCTTCCTATTTTCATGTCA--------TTTTGTATCGATCAGG  195
S. viminalis PCR T76            ---GTACGGACCGCTTCCTATTTTCATGTCA--------TTTTGTATCGATCAGG  221
S. viminalis 1112               ---GTACGGACCGCTTCCTATTTTCATGTCA--------TTTTGTATCGATCAGG  288
S. viminalis 1114               ---GTACTGGCTGCTTCCTATTTTCATGTCA--------TTTTGTATCGATCAGG  326
S. viminalis 0535 79 kbp        ---GTACTGGCTGCTTCCTATTTTCATGTCA--------TTTTGTATCGATCAGG  324
S. viminalis 0535 172 kbp       ---GTACTGGCCGCTTCCTATTTTCATGTCA--------TTTTGTATCGATCAGG  324
S. viminalis 0535 191 kbp       ---GTACTGGCTGCTTCCTATTT-CATGTCA--------TTTTGTATCGATCAGG  324
S. purpurea Chr09               ---GTACGGGCCGCTTCCTATTTTCATGTCA--------TTTTGTATCGATCAGG  252
S. purpurea Chr15W              ---GTACTGGCCGCTTCCTATTTTCATGTCA--------TTTTGTATCGATCAGG  245
S. suchowensis scaffold13213    ---GTACTGGCTGCTTCCTATTTTCATGTCA--------TTTTGTATCGATCAGG  233
S. suchowensis scaffold04567    ---GTACGGATCGCTTCCTATTTTCATGTCATTTTGACATTTTGTATCGATCAGG  248
P. trichocarpa Chr09            TACGTACGAACTGCTTAATATCTGCATGTCA--------TTTTGTATTGATCAGG  216
                                 340       350       360       370       380       390
```

```
                                 selected
S. viminalis PCR 81084           ACCAGATAA------------  204
S. viminalis PCR T76             ACCAGATAC------------  230
S. viminalis 1112                ACCAGATAATCTGCCGCTTC   308
S. viminalis 1114                ACCAGATAATCTGCCGCTTC   346
S. viminalis 0535 79 kbp         ACCAGATAATCTGCCGCTTC   344
S. viminalis 0535 172 kbp        ACCAGATAATCTGCCGCTTC   344
S. viminalis 0535 191 kbp        ACCAGATAATCTGCCGCTTC   344
S. purpurea Chr09                ACCAGATAATCTGCCGCTTC   272
S. purpurea Chr15W               ACCAGATAATCTGCCGCTTC   265
S. suchowensis scaffold13213     ACCAGATAA------------  242
S. suchowensis scaffold04567     ACCAGATA-------------  256
P. trichocarpa Chr09             AGCAGATAATCTGCCACTTC   236
                                       400       410
```

Figure 2: Multiple Sequence Alignment as used for the phylogenetic tree. Regions that where selected by Gblocks are highlighted.

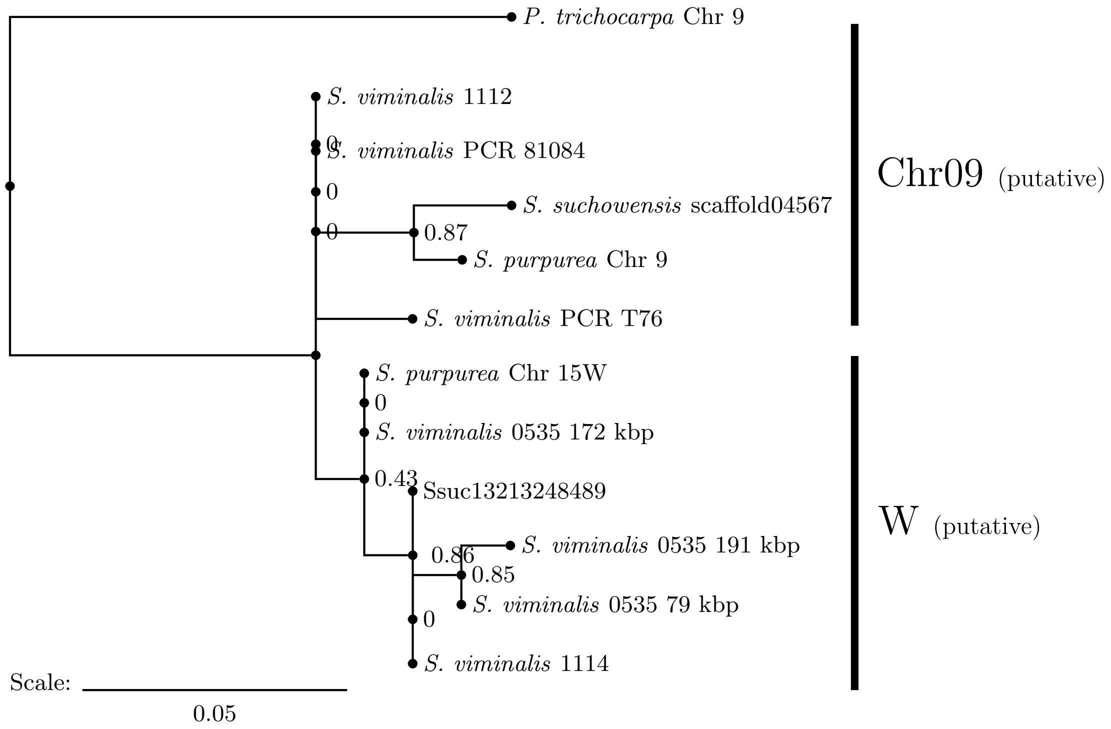

Figure 3: Phylogenetic tree of homologous sequences found in different scaffolds of the *Salix viminalis* genome assembly together with the sequence of PCR products of two male *S. viminalis* individuals (81084 and T76) as well as homologous sequence found in the *Salix pupurea* (2 sequences), *Salix suchowensis* (2 sequences) and *Populus trichocarpa* (1 sequence) genome assemblies. The male PCR products cluster together with the *S. viminalis* scaffold CAADRP01000**1112** (Chr09) and the *S. pupurea* sequence from Chr09. One of the *S. suchowensis* sequences falls in the same cluster. Sequences from the *S. viminalis* scaffolds CAADRP01000**0535** (Chr15) and CAADRP01000**1114** (previously unassigned) form a monophyletic group together with the other *S. suchowensis* scaffold and the *S. pupurea* sequence from Chr15W representing a putative W specific hemizygous region.

Reconstruction of the phylogenetic tree

The PCR products from the two male individuals were sequence using Sanger sequencing (Macrogen Inc.). Homologous sequences to the PCR products from the two male individuals were identified by BLASTn in the *S. viminalis* genome assembly[1], the *S. purpurea* genome assembly v5.1 (DOE-JGI, `https://phytozome-next.jgi.doe.gov/info/Spurpurea_v5_1`), the *S. suchowensis* genome assembly v2.0 (`http://115.29.234.170/willow`) and the *P. trichocarpa* genome assembly v4.1 (DOE-JGI, `https://phytozome-next.jgi.doe.gov/info/Ptrichocarpa_v4_1`). The analysis was performed on the Phylogeny.fr platform and comprised the following steps. Homologous sequences where aligned with T-Coffee (v11.00.8cbe486) [4] using the following pair-wise alignment methods: the 10 best local alignments (Lalign_pair), an accurate global alignment (slow_pair). After alignment, ambiguous regions (i.e. containing gaps and/or poorly aligned) were removed with Gblocks (v0.91b) [2] using the following parameters: minimum length of a block after gap cleaning: 10, no gap positions were allowed in the final alignment, all segments with contiguous nonconserved positions bigger than 8 were rejected, minimum number of sequences for a flank position: 85%. The phylogenetic tree was reconstructed using the maximum likelihood method implemented in the PhyML program (v3.1/3.0 aLRT) [3]. The default substitution model was selected assuming an estimated proportion of invariant sites (of 0.001) and 4 gamma-distributed rate categories to account for rate heterogeneity across sites. The gamma shape parameter was estimated directly from the data (gamma=95.669). Reliability for internal branch was assessed using the aLRT test (SH-Like) and the root was determined by using *P. trichocarpa* as an outgroup.

References

[1] Almeida, P., Proux-Wera, E., Churcher, A., Soler, L., Dainat, J., Pucholt, P., Nordlund, J., Martin, T., Rönnberg-Wästljung, A.-C., Nystedt, B., Berlin, S., and Mank, J. E. Genome assembly of the basket willow, salix viminalis, reveals earliest stages of sex chromosome expansion. *BMC Biology 18*, 1 (jun 2020).

[2] Castresana, J. Selection of conserved blocks from multiple alignments for their use in phylogenetic analysis. *Molecular Biology and Evolution 17*, 4 (2000), 540–552.

[3] Guindon, S., and Gascuel, O. A simple, fast, and accurate algorithm to estimate large phylogenies by maximum likelihood. *Systematic Biology 52*, 5 (2003), 696–704.

[4] Notredame, C., Higgins, D. G., and Heringa, J. T-Coffee: A novel method for fast and accurate multiple sequence alignment. *J. Mol. Biol. 302*, 1 (2000), 205–217.
